# End of Life Care on a Neuropsychiatric Inpatient Ward for Patients with Huntington's Disease: An Overview of Issues and a Project to Optimise Care

**DOI:** 10.1192/bjo.2022.336

**Published:** 2022-06-20

**Authors:** Kris Roberts, Fiona Nielsen, Maria Dale, Reza Kiani

**Affiliations:** Leicestershire Partnership NHS Trust, Leicester, United Kingdom

## Abstract

**Aims:**

Mill Lodge is a 14-bed inpatient neuropsychiatric ward in Leicestershire, UK. The service primarily functions for patients with Huntington's Disease (HD), a disorder that significantly reduces life expectancy. End of Life (EoL) care is necessitated in the inpatient setting. This project therefore aims to optimise EoL care in our specialist HD unit. Specific objectives are to: establish the levels of staff confidence in dealing with EoL care; identify specific areas of EoL care that staff felt could be improved; and to introduce a series of initiatives to optimise EoL care for our patients using a QI framework.

**Methods:**

We commenced involvement with the local QI team to develop the project. The first stage of intervention included the planning and delivery of a stakeholder event on EoL care specific to HD with the assistance of regional palliative care colleagues.

As well as our inpatient nursing and medical staff and the palliative care teams, local GPs, district nursing colleagues, speech and language therapists and psychologists attended. The session comprised an educational overview for all colleagues of HD itself and palliation was discussed at length.

The meeting also comprised an open forum where we were able to identify barriers and facilitators to optimal care from all aspects of the assembled MDT.

**Results:**

To date our interactions have revealed that staff confidence in dealing with the different aspects of EoL care was low. This included issues with care-planning; medications; communication with patients and staff; and when to refer for specialist help.

Other processes identified as difficult included paperwork that was not consistent across teams; district nursing colleagues having to liaise with multiple medical team members to ensure continuity of care; and the doses of EoL medications required in this patient group to mitigate involuntary movements that were previously controlled with multiple high-dose oral medications.

**Conclusion:**

Staff without specialist knowledge require support. The efforts made to improve collaboration with external colleagues broke down barriers that were preventing optimal care and allowed all parties to express their opinions and feelings. This allowed us to transparently appraise our current processes and provide guidance on this difficult area.

The journey of optimisation continues, with further practical educational interventions planned, such as syringe-driver training, and efforts to improve shared documentation and enhanced communication and collaborative working between different disciplines.

Optimal, collaborative EoL care from a confident staff-group is possible and a most important part of care for this unique patient group.

